# Dentists' Habits of Antibiotic Prescribing May be Influenced by Patient Requests for Prescriptions

**DOI:** 10.1155/2022/5318753

**Published:** 2022-08-22

**Authors:** Aceil Al-Khatib, Raneem Ahmad AlMohammad

**Affiliations:** Faculty of Dentistry, Jordan University of Science and Technology, P.O. Box 3030, Irbid 22110, Jordan

## Abstract

**Objective:**

This study evaluates dentists' antibiotic prescribing habits and the frequency of facing patient pressure for prescriptions.

**Methods:**

An online anonymous survey was used to collect data on antibiotic prescribing practices, including prescribing unnecessary antibiotics if requested by patients.

**Results:**

The study population included 345 dentists; 227 (65.8%) were females and 118 (34.2%) were males. 54 (15.7%) reported that they prescribed unnecessary antibiotics more than once per week, 47 (13.6%) once per month, 135 (39.1%) rarely, and 109 (31.6%) never prescribed unnecessary antibiotics. 117 (33.9%) reported being pressured by patients to prescribe unnecessary antibiotics more than once per week. 110 (31.9%) reported being pressured by patients to do so at least once per month. There was a statistical difference between the two genders (*P* < 0.001) in reporting that patients pressured them to prescribe antibiotics when antibiotics were not necessary for treatment or prophylactic purposes and in prescribing unnecessary antibiotics sometimes if requested by a patient (*P*=0.008). In addition, there was a statistical difference in dentists' confidence in their knowledge and practice in the area of antibiotic prescribing (*P* < 0.001).

**Conclusions:**

The results show that unnecessary antibiotic prescribing by dentists can be influenced by patient pressure.

## 1. Introduction

Antibiotics are frequently prescribed in the dental office, either as a treatment or as a prophylactic measure. Lack of knowledge regarding the choice of antibiotics or the indication for prescribing antibiotics is likely to lead to abuse, misuse, and unnecessary antibiotic prescribing. Inappropriate antibiotic prescribing practices have contributed to the problem of antibiotic resistance [1]. In 2021, the World Health Organization declared antimicrobial resistance to be one of the top ten global public health threats facing humanity and that misuse and overuse of antimicrobials are the main drivers in the development of drug-resistant pathogens [2].

Dealing with the global threat of antibiotic resistance [3, 4] cannot be achieved without improving antibiotic prescribing practices among all healthcare workers including dentists [5]. Therefore, evaluating the knowledge of Jordanian dentists and assessing their antibiotic prescribing practices are important to determine the factors that may contribute to inappropriate and unnecessary antibiotic prescribing.

Although recent trends in inappropriate and unnecessary prescribing of antibiotics by dentists have been documented by researchers from other countries [6–9], little is known about the current prescribing practices of dentists in Jordan and the reasons for unnecessary antibiotic prescribing.

A previous study of therapeutic adult antibiotic prescriptions by Jordanian dentists showed that dental practitioners had the tendency to prescribe antibiotics and to prescribe longer courses of antibiotics, and amoxicillin and metronidazole were the most commonly prescribed antibiotics [10]. Authors of another previous investigation looked at the problem of self-medication with antibiotics, and they postulated that physicians sometimes prescribed antibiotics for children under pressure from parents [11], an assumption that was reiterated by Al-Niemat et al. [12]. Prescribing unnecessary antibiotics under the pressure of patients has also been documented by Sawair et al. [13].

The objectives of this study were to evaluate dentists' antibiotic prescribing practices, the frequency of facing patient pressure for unnecessary prescriptions that could lead to unnecessary antibiotic prescribing, and the perceived reasons why patients may ask dentists to prescribe unnecessary antibiotics.

## 2. Methods

### 2.1. Study Design

This is a questionnaire-based cross-sectional observational study in which consenting practicing dentists completed an anonymised self-administered online questionnaire. The questionnaires were collected between January 2020 and June 2021.

### 2.2. Data Collection Instrument

An anonymous online questionnaire was created by the authors and used to evaluate antibiotic prescribing habits, reasons that contribute to unnecessary antibiotic prescribing, and dentists' opinions on the frequency of facing requests for antibiotic prescriptions by patients when antibiotics are not indicated.

The questionnaire was divided into five sections. The first section was a description of the purpose of the study and an informed consent where invited dentists indicated if they would consent to participate. The second section included questions about demographic characteristics of participants: sex, age, highest degree, practice area, years practicing dentistry, and type of employment. The third section included questions about antibiotic prescribing practices: frequency of prescribing if pressured by patients and perceived reasons why some patients request antibiotic prescriptions. The fourth section included questions about antibiotic prescribing habits, indications, the antibiotic of choice that dentists used in the treatment of patients with dental infections, the rationale for drug selection, and the duration of treatment. The fifth section's questions were about dentists' use of antibiotic prescribing guidelines, the resources dentists use to inform their decisions, and their confidence in their knowledge about antibiotic prescribing.

### 2.3. Development of the Questionnaire

Before distributing the questionnaire to practicing dentists, questions were tested for face and content validity by asking qualified experts to review the items of the English questionnaire, these experts were asked for suggestions to improve the questions, and ambiguous or confusing questions were deleted or revised and amended. Then, the questionnaire was pilot tested for comprehensibility, and to determine the time required to fill out the questionnaire, pilot testing was achieved by administering the questionnaire to a sample of 20 dentists who were excluded from the study. Testing and retesting of the questionnaire were undertaken; revisions and corrections were made when needed before drafting and distributing the final version.

### 2.4. Data Collection and Management

A Google Form was prepared to distribute the final version of the questionnaire to participating dentists. The questionnaire's link was posted on Facebook (group and personal account) and sent via dentists' WhatsApp groups. Potential respondents were asked to share the questionnaire's link with other dentists.

### 2.5. Ethical Considerations

The study was approved by the Institutional Review Board (IRB) of King Abdullah University Hospital (KAUH), Irbid, Jordan (Ref.: 114/118/2018).

### 2.6. Statistical Analysis

The collected anonymised data were analysed by Microsoft Office Excel 2013 (Microsoft Corporation, Redmond, Washington) and by the Statistical Program for Social Sciences IBM SPSS Statistics version 22 (IBM SPSS Statistics for Windows, Version 22.0. IBM Corp., Armonk, NY). The association between variables was determined by the chi-squared test or Fisher's exact test. The statistical significance level was set at *P* < 0.05. For the purpose of this study, the analysis included responses to questions on the respondents' characteristics, the number of years each respondent had been practicing dentistry, the frequency of prescribing unnecessary antibiotics, exposure to patients' pressure to prescribe antibiotics, dentists' confidence in knowledge in the area of antibiotic prescribing, and dentists' prescribing practices.

## 3. Results

### 3.1. Respondents' Characteristics

The study population included 345 dentists; 227 (65.8%) were females and 118 (34.2%) were males with a mean age of 29.7 years (SD, 8.6 years). 69.3% were general dentists. 68.1% had been practicing dentistry for less than 5 years (Table 1).

### 3.2. Reported Frequency of Prescribing Unnecessary Antibiotics

54 (15.7%) of the respondents reported they prescribed unnecessary antibiotics more than once per week, 47 (13.6%) once per month, 135 (39.1%) rarely, and 109 (31.6%) never prescribed unnecessary antibiotics.

### 3.3. Reported Exposure to Patients' Pressure to Prescribe Antibiotics

**Figure 1:**
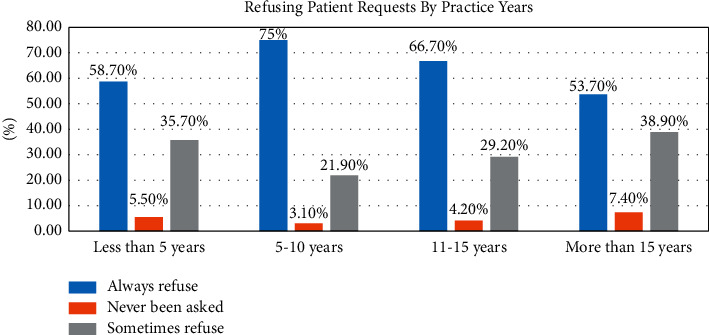
Percentage of responses denying patient requests for unnecessary antibiotics.

**Figure 2:**
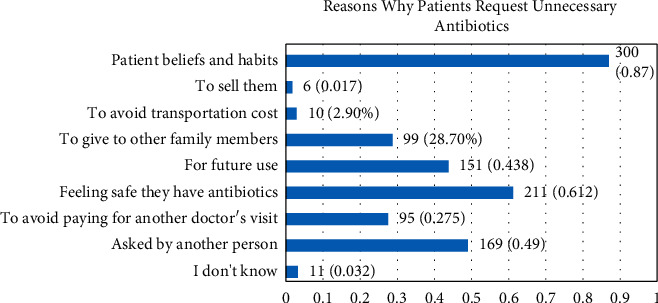
Percentage of responses selected by dentists as possible reasons why patients may ask dentists to prescribe unnecessary antibiotics (dentists could select more than one choice).

117 (33.9%) reported being pressured by patients to prescribe unnecessary antibiotics more than once per week. 110 (31.9%) reported being pressured by patients to do so at least once per month. There was a statistical difference between the two genders (*P* < 0.001) in reporting pressure by patients to prescribe antibiotics when they were sure that antibiotics were not necessary for treatment purposes (Table 2) and prophylactic purposes (Table 2). In addition, there was a gender difference in prescribing unnecessary antibiotics sometimes if requested by a patient (*P*=0.008).  Figure 1 shows the association between the frequency of refusing patient requests for antibiotic prescriptions and the number of years in dental practice.  Figure 2 illustrates dentists' perspectives on why patients may request antibiotic prescriptions when antibiotic treatment is not indicated.

### 3.4. Reported Confidence in Knowledge and Prescribing Practices

**Figure 3:**
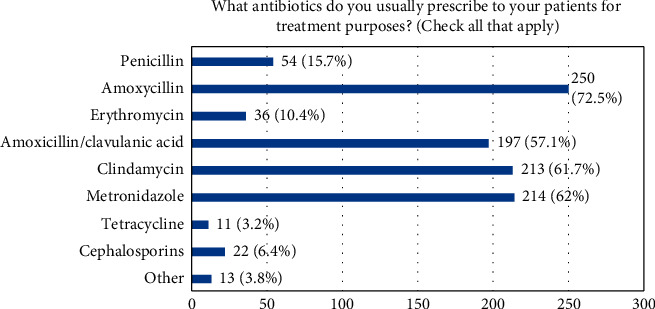
Antibiotics prescribed by dentists for treatment purposes.

53.6% of the study population (72.0% of males and 44.1% of females; *P* < 0.001) felt confident about their knowledge and practice in the area of antibiotic prescribing.  Table 3 shows the systemic diseases and conditions for which dentists reported prescribing antibiotics prophylactically, the procedures before which dentists prescribe antibiotics, the reasons dentists prescribe antibiotics, and the duration of antibiotics used when indicated. The majority of respondents selected amoxicillin, metronidazole, clindamycin, and amoxicillin/clavulanic acid as the antibiotics they usually prescribe for treatment purposes as shown in Figure 3.

## 4. Discussion

Antibiotics are recommended in the treatment of odontogenic and nonodontogenic infections when there are signs of spread of infection or systemic involvement [14]. Antibiotics are also necessary for prophylactic purposes to prevent endocarditis in high-risk patients [15], to prevent surgical site infections [16], and to prevent prosthetic joint infection in a subset of patients with artificial joints [17]. Overprescribing antibiotics occurs when antibiotics are used to treat infections that can be controlled by local interventions in patients who are not at high risk to develop complications. Antibiotic overprescribing also occurs when patients with uncomplicated cellulitis receive a prolonged course of therapy [18, 19]. Based on recent studies and current guidelines, antibiotics should not be routinely used prior to performing the following dental procedures: fillings, simple root canal treatment (RCT), RCT with localized abscess, RCT with a sinus tract, tooth preparation for a fixed prosthesis, scaling and polishing, placing orthodontic brackets, administration of routine anaesthesia, or taking radiographs.

It is important to bear in mind that overprescribing antibiotics is associated with long-term risks including life-threatening diseases, opportunistic infections like *Clostridium difficile*, increased adverse events, increased risk of complications, increased mortality rate, increased hospital admissions, and increase in antibiotic resistance [20]. Dentists and patients have an important role to play in the fight against antibiotic resistance, and it is thus important to understand dentists' habits in antibiotic prescribing and the impact of patient requests for antibiotic prescriptions on those habits.

Therefore, this study was performed to investigate, anonymously, the habits of dentists in antibiotic prescribing. Findings from this study add to the corpus of the literature on the factors that contribute to the misuse and overuse of these medications and provide insight and knowledge surrounding the habits and frequency of prescribing, which is essential to combat the global threat of bacterial resistance. To the best of our knowledge, this might be one of the first recent studies that provide an assessment of dentists' exposure to patients who request prescriptions and their perception of why patients request unnecessary antibiotics. Findings showed that 39% of surveyed dentists reported that they rarely prescribed antibiotics. In comparison, other studies have shown that dental professionals were among the highest to prescribe antibiotics. In the United States, dentists were the third highest prescribers of antibiotics in the nation by volume [7], and they were responsible for 10% of all antibiotic prescriptions in England [21].

Dentists often find themselves in a situation where the patient is convinced that a pulpal or periodontal infection will only heal if an antibiotic is used, many patients will start antibiotic treatment before getting a consultation, and findings from this study showed that 33.9% of participants reported they were frequently pressured by patients, more than once per week, to prescribe antibiotics when antibiotics were not necessary, while 22% reported that parents pressured them to prescribe unnecessary antibiotics for their children. The majority of our participants (60%) reported that they would always refuse patients' requests to prescribe unnecessary antibiotics. The relatively high percentage of patients demanding antibiotic prescriptions when it is not indicated should raise an alarm against antibiotic abuse by patients, should ring a bell for public health stakeholders, and serve as a call for more focus on implementing education programs aimed at raising awareness about the harm of patients self-administering antibiotics. An interesting finding was that cash payers (patients without insurance) were less likely to ask or pressure their dentists to prescribe antibiotics when antibiotics were not necessary, and this finding highlights the potential role of insurance companies in controlling the problem of unnecessary antibiotic prescribing.

Analyzing the current guidelines in terms of antibiotic prophylactic prescription for the prevention of infective endocarditis reveals differences but indicates that there are currently relatively few patient subpopulations for which antibiotic prophylaxis may be indicated prior to certain dental procedures [22–24].

In this study, a history of infective endocarditis was reported to be the most common cause for prophylactic antibiotic prescriptions by 74.8.% of the respondents, followed by the presence of a prosthetic heart valve for which 73.3% of the surveyed dentists prescribed antibiotic prophylaxis. These findings are similar to results reported in other populations. For example, in the US, 70% of dentists reported having had patients who took antibiotics before a dental procedure for the prevention of infective endocarditis [25], and in a study by Jain et al. [26], the authors reported that some dentists failed to prescribe prophylactic antibiotics for patients who were at risk. According to the recent update of the 2008 NICE guidelines, antibiotic prophylactic is no longer recommended routinely to prevent infective endocarditis [27] in patients undergoing dental procedures. On the other hand, the American Heart Association (AHA) and the European Society of Cardiology (ESC) guidelines suggest antibiotic prophylaxis for patients with a previous relapse or recurrent infective endocarditis and for patients with a prosthetic heart valve. The finding that some dentists do not agree on whether to or not to prescribe prophylactic antibiotics in high-risk patients confirms a recent finding by [28] who concluded that antibiotic prescribing practices were inconsistent, and dentists' practices did not meet the highest antibiotic stewardship standards. Furthermore, findings from this study highlight the lack of consensus in the guidelines and perhaps the confusion surrounding this area as suggested by [29] and Lagha et al. [30]. This further suggests that the majority of Jordanian dentists follow the European [22] and American guidelines [24].

Regarding antibiotic prophylaxis for patients with prosthetic joints, the American Dental Association's (ADA) 2015 guidelines state: “In general, for patients with prosthetic joint implants, prophylactic antibiotics should not be given prior to dental procedures to prevent prosthetic joint infection,” and patient's preferences should be considered. In 2013, the ADA and the American Academy of Orthopaedic Surgeons (AAOS) codeveloped an evidence-based clinical practice guideline that limited the use of antibiotic prophylaxis to high-risk patients [31, 32]. Nevertheless, although most experts do not routinely recommend the prophylactic use of antibiotics in patients with joint prostheses undergoing dental procedures [33], 29.9% of dentists who responded to this study reported prescribing antibiotic prophylaxis for patients with prosthetic joints.

Findings from this study showed that amoxicillin was the most commonly prescribed antibiotic as reported by (72.5%) of dentists, followed by metronidazole, clindamycin, and amoxicillin/clavulanic acid. This finding confirms previous findings by many studies that have reported amoxicillin as one of the most common antibiotics used in dental practice and primary care settings [34–36]. In Belgium, 82% of the antibiotic prescriptions were amoxicillin [36]. In a study conducted in Saudi Arabia, amoxicillin was also the most commonly prescribed antibiotic with a percentage of 73.8% [37]. Durkin et al. [7] reported that amoxicillin was also reported to be the most commonly prescribed antibiotic among all dental specialists in the United States in 2015. Interestingly, it was reported that amoxicillin was prescribed more frequently in England and Scotland than in Norway and Sweden from 2010 to 2016 [38]. Curiously, in an earlier study, it was reported that in Norway, the use of narrow-spectrum antibiotics was preferred as a conservative approach to medication prescribing, where the most commonly prescribed antibiotic was phenoxymethylpenicillin [39]. The reason why dentists most commonly prescribe this antibiotic may be due to the fact that this antibiotic covers a wide range of Gram-positive bacteria and also provides some coverage against Gram-negative bacteria compared to other antibiotics. It is not clear, however, if the choice of prescribing a narrow-spectrum or a broad-spectrum antibiotic to treat dental infections is population-specific [40] or has changed over time.

Dentists should be cognizant that long courses of antibiotics result in the emergence of antibiotic resistance and may complicate the treatment [41]. In this regard, findings showed that 39.4% of participants reported that the prescription duration would depend on the type of antibiotics; 33% reported the duration of 5 days, while 22.9% prescribed antibiotics for 7 days. This finding is in agreement with previous studies; for example, the length of antibiotic treatment recommended by most US dentists was seven days. These findings are in line with the duration of treatment recommended in therapeutic guidelines, which were based on expert opinions [42, 43]. A study by Yingling et al. [44] reported an average duration of antibiotic therapy of 7.58 days. Previously, studies that were performed in the Mediterranean region found that there was a trend to use lower dosages of antibiotics over a longer duration of time [10, 45].

### 4.1. Limitations of the Study and Future Recommendation

The topic of antibiotic prescribing is complex and wide, and dentists' knowledge of antibiotic prescribing cannot be evaluated objectively. The current study was a survey-type investigation that used social media platforms to distribute the questionnaire's link, and it has some limitations including its limitation in determining the response rate and selection bias because it is fairly difficult to know how many dentists received the questionnaire and how many dentists accurately reported their prescribing habits.

The frequency of antibiotic prescribing by dentists in comparison to the prescribing habits of other healthcare providers was not carried out and is worthy of investigation.

## 5. Conclusions

This study indicates that antibiotic prescribing by dentists is not consistent with the current guidelines. A significant number of respondents reported prescribing clindamycin for treatment purposes, and a minority reported that they routinely prescribe unnecessary antibiotics for common dental procedures. In addition, findings show that unnecessary antibiotic prescribing by dentists can be influenced by patients' pressure. Findings from this study provide a heuristic approach to identifying some of the factors and solutions to the problem of misuse and abuse of antibiotics in dental practice. Some solutions include updating and clarifying the guidelines, restricting antibiotic use to cases where the benefit outweighs the risk of harm, prohibiting over-the-counter antibiotic availability, educating dentists on how to respond to patients' requests for antibiotic prescriptions in order to minimize the use of antibiotics when they are not necessary, which is an important intervention strategy for addressing the challenge of bacterial resistance, and implementing antibiotic stewardship practices and programs.

## Figures and Tables

**Table 1:** Respondents' characteristics.

Characteristics	Count	Percentage
Gender		
Female	277	65.8%
Male	118	34.2%

Highest degree/professional certification received		
Bachelor of Dental Surgery	239	69.3%
Advanced degrees and postgraduate training	106	30.7%

Practice area		
General dentistry	239	69.3%
Oral and maxillofacial surgery	18	5.2%
Periodontics	17	4.9%
Orthodontics	9	2.6%
Pediatric dentistry	16	4.6%
Prosthodontics	8	2.3%
Conservative/operative dentistry	9	2.6%
Endodontic	23	6.7%
Oral medicine	6	1.7%

Years practicing dentistry		
Less than 5 years	235	68.1%
5–10 years	32	9.3%
11–15 years	24	7%
More than 15 years	54	15.7%

Employment		
A full-time dentist in his/her private clinic	157	45.5%
An academic at a university where they teach and see patients	17	4.9%
A full-time dentist at a military's royal medical service hospital	26	7.5%
A full-time dentist at the ministry of health hospital	26	7.5%
A full-time dentist at the ministry of health centre	25	7.2%
A part-time dentist	94	27.2%

**Table 2:** Unnecessary antibiotic prescribing by gender.

	Responses	Females (%)	Males (%)	*P* value
How often do you prescribe antibiotics when you are sure they are not necessary?	More than once per week	14.1	18.6	0.601
	Never	31.7	31.4	
	Once per month	15.0	11.0	
	Rarely	39.2	39.0	

How often do patients pressure you to prescribe antibiotics when you are sure they are not necessary for treatment or prophylactic purposes?	At least once per month	39.2	17.8	<0.001
	More than once per week	31.3	39.0	
	Never	6.2	13.6	
	Rarely	23.3	29.7	

^∗^How often do patients pressure you to prescribe antibiotics for their children when you are sure antibiotics are not necessary?	At least once per month	31.3	20.3	0.217
	More than once per week	21.6	22.9	
	Never	11.5	16.1	
	Rarely	25.6	27.1	

Do you prescribe unnecessary antibiotics if requested by the patient?	Always	0.4	2.5	0.008
	Never	55.9	60.2	
	Rarely	26.9	31.4	
	Sometimes	16.7	5.9	

Have you ever refused to prescribe antibiotics if the patient asked you to prescribe unnecessary antibiotics?	I always refuse	58.1	63.6	0.554
	Never been asked	5.3	5.9	
	I sometimes refuse	36.6	30.5	

**Table 3:** Dentists' antibiotics prescribing habits.

Prophylactic antibiotics with surgical procedures	Diabetes	111	32.2%
	Congenital heart disease	147	42.6%
	Mitral valve prolapse without regurgitation	104	30.1%
	Mitral valve prolapse with regurgitation	168	48.7%
	Prosthetic heart valve	253	73.3%
	History of infective endocarditis	258	74.8%
	History of myocardial infarction	73	21.2%
	Coronary artery stents	91	26.4%
	Any heart problem	40	11.6%
	Artificial joints	103	29.9%
	Patients on steroid therapy	59	17.1%
	If patient requests	8	2.3%
	Fillings	4	1.2%
	Simple root canal treatment	13	3.8%
	Root canal treatment with a localized abscess	100	29%
	Root canal treatment with the sinus tract	78	22.6%
	Root canal treatment with cellulitis	237	68.7%
	Surgical extractions	195	56.5%
	Simple extractions	42	12.2%
	Placing dental implants	147	42.6%
	Tooth preparation for a fixed prosthesis	5	1.4%
	Root planning	52	15.1%
	Scaling and polishing	20	5.8%
	Periodontal surgery	110	31.9%
	Placing orthodontic brackets	2	0.6%
	Administration of routine anesthesia	4	1.2%
	Taking radiographs	2	0.6%

Prescribing antibiotics for patients who do not have a systemic disease	To prevent infective endocarditis	63	18.3%
	To prevent infection in the operation area	77	22.3%
	To prevent septicemia	35	10.3%
	To decrease postoperative inflammation	60	17.4%
	To decrease postoperative pain	21	6.1%
	When sterilization is inadequate	54	15.7%
	To prevent osteomyelitis	20	5.8%
	To prevent dry socket	14	4.1%
	To treat dry socket	23	6.7%
	To prevent secondary infection	29	8.4%
	Distrust infection control measures	15	4.3%
	To please the patient	6	1.7%
	Do not prescribe antibiotics to healthy patients	187	54.2%

Duration of antibiotic treatment prescribed when indicated	3 days	14	4.1%
	5 days	114	33%
	7 days	79	22.9%
	10 days	2	0.6%
	Depends on the antibiotic being prescribed	136	39.4%

## Data Availability

The data used to support the findings of this study are available from the corresponding author upon request.
